# Maternal near-miss and death and their association with caesarean section complications: a cross-sectional study at a university hospital and a regional hospital in Tanzania

**DOI:** 10.1186/1471-2393-14-244

**Published:** 2014-07-23

**Authors:** Helena Litorp, Hussein L Kidanto, Mattias Rööst, Muzdalifat Abeid, Lennarth Nyström, Birgitta Essén

**Affiliations:** Department of Women’s and Children’s Health, International Maternal and Child Health (IMCH), Uppsala University, 751 85 Uppsala, Sweden; Department of Obstetrics and Gynaecology, Muhimbili National Hospital, Kallenga street, Upanga, PO Box 65439, Dar es Salaam, Tanzania; Department of Public Health and Clinical Medicine, Epidemiology and Global Health, Umeå University, 901 85 Umeå, Sweden

**Keywords:** Maternal near-miss, Maternal death, Caesarean section, Low-income country

## Abstract

**Background:**

The maternal near-miss (MNM) concept has been developed to assess life-threatening conditions during pregnancy, childhood, and puerperium. In recent years, caesarean section (CS) rates have increased rapidly in many low- and middle-income countries, a trend which might have serious effects on maternal health. Our aim was to describe the occurrence and panorama of maternal near-miss and death in two low-resource settings, and explore their association with CS complications.

**Methods:**

We performed a cross-sectional study, including all women who fulfilled the WHO criteria for MNM or death between February and June 2012 at a university hospital and a regional hospital in Dar es Salaam, Tanzania. Cases were assessed individually to determine their association with CS. Main outcome measures included MNM ratio; maternal mortality ratio; proportion of MNM and death associated with CS complications; and the risk for such outcomes per 1,000 operations. The risk ratio of life-threatening CS complications at the university hospital compared to the regional hospital was calculated.

**Results:**

We identified 467 MNM events and 77 maternal deaths. The MNM ratio was 36 per 1,000 live births (95% CI 33–39) and the maternal mortality ratio was 587 per 100,000 live births (95% CI 460–730). Major causes were eclampsia and postpartum haemorrhage, but we also detected nine MNM events and five deaths from iatrogenic complications. CS complications accounted for 7.9% (95% CI 5.6–11) of the MNM events and 13% (95% CI 6.4–23) of the maternal deaths. The risk of experiencing a life-threatening CS complication was three times higher at the regional hospital (22/1,000 operations, 95% CI 12–37) compared to the university hospital (7.0/1,000 operations, 95% CI 3.8–12) (risk ratio 3.2, 95% CI 1.5–6.6).

**Conclusions:**

The occurrence of MNM and death at the two hospitals was high, and many cases were associated with CS complications. The maternal risks of CS in low-resource settings must not be overlooked, and measures should be taken to avoid unnecessary CSs. More comprehensive training of staff, improved postoperative surveillance, and a more even distribution of resources within the health care system might reduce the risks of CS.

**Electronic supplementary material:**

The online version of this article (doi:10.1186/1471-2393-14-244) contains supplementary material, which is available to authorized users.

## Background

Complications during pregnancy and childbirth remain a leading cause of critical illness and death among women of reproductive age in many low-income countries [1]. In recent years, the concept of maternal near-miss (MNM) has received growing attention as a way to assess severe maternal morbidity potentially leading to death. According to the World Health Organization (WHO), a MNM refers to “a woman who almost dies but survives a complication during pregnancy, childbirth, or within 42 days after termination of pregnancy” [2]. Until recently, different criteria have been used to define MNM [3]. In 2009, the WHO presented new criteria to define MNM in order to facilitate comparisons between different studies [2]. By representing aspects of organ dysfunction, the new criteria reflects true critical illness. Thus far, few published studies have applied the new criteria [4–8], and only three have been done in low-income countries [9–11].

In recent decades, caesarean section (CS) rates have increased rapidly worldwide, including in many low- and middle-income countries [12]. The causes behind this rise are not fully known, nor are the effects on maternal health, especially in low-resource settings where safety of the procedure is lower [13] and there is a lack of human and material resources [14]. We recently conducted a study at a university hospital in Tanzania that revealed a sharp increase in CS rates between 2000 and 2011, and found the rise was accompanied by a significant increase in the maternal mortality ratio (MMR) [15]. Several studies have reported increased risk of blood transfusion [16, 17], hysterectomy [16, 17], bleeding complications [18], infections [18], and maternal death [13, 16, 17, 19] among women undergoing CS. However, assessing the risks of CS versus vaginal birth is complicated, as adverse outcomes after CS might be confounded by the medical condition that lead to the operation. There are few publications with ideal study design to establish the causal relationship between CS and maternal morbidity and mortality [17, 19, 20].

Our aim was to describe the occurrence and panorama of MNM and death at a university hospital and a regional hospital in Tanzania, and explore their association with CS complications. We sought to provide valid estimates of the proportion of MNM and death directly attributed to CS complications and the risk for such outcomes per 1,000 operations.

## Methods

### Study design and data collection

We conducted a cross-sectional study at one university hospital and one regional hospital in Dar es Salaam, Tanzania, between February and June 2012. Obstetric and gynaecological wards were visited every second day by the main researcher (HL) and all medical records of admitted patients were reviewed in order to identify cases. The record books in which midwives document severe cases were also examined. Data on demographic and clinical characteristics were collected from medical records and antenatal cards. Maternal death files, routinely gathered by hospital staff, were reviewed monthly. Cases in which the underlying cause of MNM or death was unclear were discussed between three of the authors (HL, HK, and MA) and guidance was sought in the International Statistical Classification of Diseases and Related Health problems-Maternal Mortality [21]. As the exact chain of events was sometimes difficult to follow due to a lack of information from referring institutions, the leading cause of MNM or death was considered to be the diagnosis that most likely had put the woman in a life-threatening condition. Our definition thereby deviated from the international classification system of maternal deaths, where the underlying cause is defined as the disease or condition that initiated the morbid chain of events leading to a woman’s death [21]. Data on total number of deliveries, live births, and CSs were derived from the obstetric database at the university hospital and the birth register at the regional hospital.

### Setting

Tanzania is a low-income country with high maternal and perinatal mortality [22]. It is the policy of the government to provide maternity care free of charge. The country’s latest Demographic and Health Survey estimated the total fertility rate to 5.4 children per woman; the national CS rate to 5.0%; and the MMR to 454 maternal deaths per 100,000 live births (95% confidence interval [CI] 353–556) [22]. Due to a shortage of qualified medical doctors, Tanzania has been training non-physician clinicians, so-called assistant medical officers, since the 1960s [23]. These are secondary-school graduates who receive a total of five years of medical education, which allows them to make diagnoses, write prescriptions, and practise medicine, surgery, and anaesthesiology [23].

Dar es Salaam is the largest city in the country, with an estimated four million inhabitants [24]. Most residents live within ten kilometres of a health care facility, and 90% of all deliveries are attended by skilled personnel [22]. The public hospitals in Dar es Salaam include one university hospital, which serves as a teaching and referral institution, three regional hospitals, and one military hospital. The health care system has a hierarchical structure, where the majority of deliveries take place at health centres and regional hospitals. After an upgrade of the peripheral hospitals in the in the early 21st century, access to CSs has increased at these facilities. There are, however, still large discrepancies in the CS rates between the university hospital and the peripheral hospitals. In order to understand the MNM panorama on different levels, we conducted our study at the university hospital and in one of the regional hospitals.

As the largest public hospital in the country, the university hospital handles about 9,000 deliveries annually. The obstetric department is well-staffed, with one specialist obstetrician, two residents, and one intern doctor on call each day. Patients with critical conditions are admitted to the Eclampsia Ward, where their vital signs are monitored hourly. In the main intensive care unit, treatment with vaso-active drugs and ventilation can be provided. Blood for transfusions is supplied through the hospital’s blood bank, but is sometimes insufficient and must be supplemented by the National Blood Bank. The CS rate in 2011 was 49% and instrumental deliveries constituted around 1% of the total deliveries [15]. The majority of CSs are performed by residents (medical doctors doing their specialist training) in obstetrics and gynaecology in one of the department’s two own operating theatres. Anaesthesia is provided by nurse anaesthetic assistants (qualified nurses trained in anaesthesia) or residents in anaesthesia. There are a few licensed anaesthesiologists, who mainly work as supervisors.

The regional hospital is situated in the outskirts of Dar es Salaam. With regard to obstetric population and available resources, it is representative of the other two regional hospitals in the area. About 20,000 deliveries are performed annually. During the study period, two specialists in obstetrics and gynaecology, seven registrars (medical doctors working after completing their internship but before starting specialist training), and eleven assistant medical officers worked in the obstetric and gynaecological wards. There is a conspicuous shortage of equipment, including gloves, syringes, Oxytocin, and electricity. Laboratory services are rarely available. The Eclampsia Ward admits patients with eclampsia and other severe conditions. Magnesium sulphate is usually in stock. Blood for transfusions is provided by the National Blood Bank, which allocates a few units to the hospital every day. As there is only one operating theatre serving the entire hospital, the facilities cannot meet the demands for CS and patients are occasionally referred to the university hospital for surgery. CSs are performed by registrars or assistant medical officers. Anaesthesia is provided by nurse anaesthetic assistants or assistant medical officers.

### Participants

We included MNM events based on the WHO criteria [2, 25] and maternal deaths according to the WHO definition [21] among all women with complications during pregnancy, childbirth, or within 42 days after termination of pregnancy. A near-miss criterion was considered fulfilled if stated in the medical record or if it could be observed by the researcher, e.g. hyperventilation, repeated fits, or jaundice in the presence of pre-eclampsia. Due to limited resources, some laboratory- and management-based criteria were not applicable. As we hoped to include patients on the clinical criteria and wanted to make results as comparable as possible with other studies, we did not modify the criteria. The definitions of the criteria, their applicability in the two settings, and how we interpreted them are presented in Table A1, Additional file 1. For example, we interpreted the criterion “uncontrollable fits” as unconsciousness and repeated fits. We followed women during hospitalization until their discharge or death. Once women were discharged, they were considered to have survived. Women who were re-admitted to one of the study sites within 42 days after termination of pregnancy and died, were recorded as maternal deaths. Referrals from the regional hospital to the university hospital were presented in the data for the university hospital. Women who experienced two unrelated MNM events, such as eclampsia and infection, were recorded as two events.

In order to identify women who had experienced a MNM event or death due to a CS complication, we assessed the files of all women who had fulfilled their first MNM criterion or died after having a CS, or had a diagnosis that implied a CS complication. All cases potentially associated with CS were reviewed by four of the authors (HL, KH, MR, and BE) to reach a consensus on whether they were associated with the CS or not. In the assessment, the indication of CS, the timing of MNM or death, and any pre-existing conditions were taken into account. The association between MNM or death and CS was graded as strong, moderate, or weak. Strong associations were complications specific to surgery or anaesthesia, for example damage to intra-abdominal organs. Complications not specific to surgery or anaesthesia, but with an increased risk after CS (e.g. postpartum haemorrhage leading to shock, hysterectomy, blood transfusion, or death [13, 16–18]), were considered moderate associations. Moderate associations also included cases where there was a pre-existing condition that might have affected the outcome, such as severe pre-eclampsia predisposing the woman to intra-abdominal haemorrhage after CS. Weak associations were cases in which it was unlikely that the CS complication itself had caused the MNM event or death.

### Analysis

Data was computerised using Excel and analysed with SPSS. We calculated the MNM ratio (MNMR), defined as the number of MNM events per 1,000 live births, and the MMR, defined as the number of maternal deaths per 100,000 live births. Since many patients at the university hospital had been referred after being delivered at other hospitals, we also calculated the MNMR and MMR for women delivered only at the university hospital and only at the regional hospital, excluding women delivered elsewhere. The mortality index was calculated by dividing the number of maternal deaths by the sum of MNM events and maternal deaths [25].

The proportion of MNM and death attributed to CS complications was calculated by dividing the number of MNM events and deaths with strong or moderate association with CS by the total number of MNM events and deaths at the two hospitals. To estimate the risk of CS complications per 1,000 operations, we divided the number of MNM events and deaths with strong or moderate association with CS by the total number of CSs at the two facilities. The risk ratio of life-threatening CS complications at the university hospital compared to the regional hospital was also calculated. For all estimates, we computed the 95% CI.

### Ethics approval

Clearance to conduct the study was obtained from the Ethics Board at Muhimbili University for Health and Allied Sciences (reference number MU/RP/AEC/Vol. XIII) on 23 December 2011. A research permit was given by the Tanzania Commission for Science and Technology (reference number 2012-39-NA-2011-191) on 17 February 2012. Permission to collect data was obtained from the administrations at Muhimbili National Hospital and Temeke Hospital. Informed consent from patients to use the information was not obtained. Data entered into the database was coded and rendered anonymous as to patient identity.

## Results

We identified 467 MNM events and 77 maternal deaths among 13,121 live births. Ten women experienced two separate MNM events. Discharge or death occurred in all cases within 42 days after termination of pregnancy. Information on time of discharge was missing in 28 cases. Because we regarded it as likely that those women had been discharged alive, they were included as MNM cases.

The characteristics of women with MNM events and deaths are presented in Table 1. Most MNM events and deaths occurred in third trimester (42%) or puerperium (40%). Eighty-seven percent (n = 326) of the MNM events and deaths at the university hospital and 11% (n = 19) at the regional hospital were referrals from other institutions. Among MNM events and deaths, labour was induced in 14% (n = 54) at the university hospital and 27% (n = 45) at the regional hospital. Only a few women (2.9%) who later developed MNM events or died had a chronic disease or previous surgery (excluding CS) noted in their medical record. Most women (88%) had visited an antenatal clinic. Among them, hypertension or pre-eclampsia were detected in 16% (n = 77). No antenatal problems were detected in 77% (n = 370) of the women.

**Table 1:** **Characteristics of maternal near-misses and deaths at a university hospital and a regional hospital in Tanzania between February and June 2012**

Characteristic	University hospital	Regional hospital	Total
	(n = 374)	(n = 170)	(n = 544)
Maternal age (years)			
Mean (SD)	27 (7)	25 (6)	26 (7)
Range	14–48	15–41	14–48
Missing	1 (0.3%)	3 (1.8%)	4 (0.7%)
Parity			
0	145 (39%)	88 (52%)	233 (43%)
1–4	202 (54%)	69 (41%)	271 (50%)
> 4	18 (4.8%)	3 (1.8%)	21 (3.9%)
Missing	9 (2.4%)	10 (5.9%)	19 (3.5%)
Area of residence			
Urban	175 (47%)	7 (4.1%)	182 (34%)
Semi-urban	101 (27%)	121 (71%)	222 (41%)
Rural	80 (21%)	35 (21%)	115 (21%)
Missing	18 (4.8%)	7 (4.1%)	25 (4.6%)
Education			
None	33 (8.8%)	13 (6.4%)	46 (8.5%)
Primary	205 (55%)	76 (45%)	281 (52%)
Secondary or higher	71 (19%)	17 (10%)	88 (16%)
Missing	65 (17%)	64 (38%)	129 (24%)
Marital status			
Cohabiting with partner	305 (82%)	107 (63%)	412 (76%)
Not cohabiting with partner^a^	19 (5.1%)	7 (4.1%)	26 (4.8%)
Missing	50 (13%)	56 (33%)	106 (20%)
Previous caesarean section			
Yes	52 (14%)	6 (3.5%)	58 (11%)
No	322 (86%)	164 (96%)	486 (89%)
HIV status			
Positive	35 (9.4%)	11 (6.5%)	46 (8.9%)
Negative	269 (72%)	87 (51%)	356 (65%)
Missing	70 (19%)	72 (42%)	142 (26%)
Termination of pregnancy			
Abortion < 28 weeks gestation^b^	33 (8.8%)	14 (8.2%)	47 (8.6%)
Laparatomy for ectopic pregnancy	18 (4.8%)	15 (8.8%)	33 (6.1%)
Vaginal delivery^c^	132 (35%)	82 (48%)	214 (39%)
Caesarean section	168 (45%)	21 (12%)	189 (35%)
Died pregnant	8 (2.1%)	13 (7.6%)	21 (3.9%)
Discharged pregnant	4 (1.1%)	2 (1.2%)	6 (1.1%)
Missing	11 (2.9%)	23 (14%)	34 (6.3%)

^a^Single, widowed, or divorced.

^b^Includes complete spontaneous abortion, incomplete spontaneous abortion terminated with curettage, and unsafe abortion.

^c^Pregnancies with ≥ 28 weeks gestation.

The most common criteria fulfilled by women experiencing MNM events were fits (35%), shock (24%), and hysterectomy (10%) (Table A2, Additional file 1). Maternal near-miss criteria were fulfilled on arrival in 43% and after arrival in 56%, with no large differences between the university hospital and the regional hospital. Eighty-two percent (180/222) of women with eclampsia met the MNM criteria already on arrival, and of these 106 women had been referred from other institutions and 74 came from home. On the contrary, most women with uterine rupture (71%) met their first criterion during hospital stay.

The university hospital had a higher MNMR and MMR than the regional hospital, but a lower mortality index (13% vs. 18%) (Table 2).

**Table 2:** **Maternal near-miss ratio (MNMR) per 1,000 live births and maternal mortality ratio (MMR) per 100,000 live births at a university hospital and a regional hospital in Tanzania between February and June 2012, including 95% confidence intervals**

	University hospital	Regional hospital	Total
Total deliveries	3,790	9,794	13,584
Total live births	3,555	9,566	13,121
Total MNM events	326	141	467
Total maternal deaths	48	29	77
MNMR			
Included at the hospital	92 (82–102)	15 (12–17)	36^a^ (33–39)
Delivered at the hospital^b^	67 (59–75)	17 (14–20)	30 (27–33)
MMR			
Included at the hospital	1350 (1,000–1,790)	303 (200–440)	587 (460–730)
Delivered at the hospital^b^	647 (410–970)	366 (250–510)	442 (340–570)
Mortality index^c^	13% (9.6–17)	18% (13–25)	14% (11–17)

^a^When excluding second event among women with two separate MNM events, MNMR was 35 per 1,000 live births.

^b^Excluding women delivered at other hospitals.

^c^Maternal deaths divided by the sum of MNM events and deaths.

Major causes of MNM were hypertensive disorders and postpartum haemorrhage (Table 3). Most deaths were caused by eclampsia, ablatio placenta, and peripartum cardiomyopathy. We also detected cases with iatrogenic complications, including two MNM events due to uterine perforation following curettage, one death from magnesium intoxication, one death from a blood transfusion reaction, and complications related to CS.

**Table 3:** **Causes of maternal near-miss (MNM) and death at a university hospital and a regional hospital in Tanzania between February and June 2012**

	University hospital		Regional hospital		
Cause of MNM or death	MNM	Death	MNM	Death	Total
	(n = 326)	(n = 48)	(n = 141)	(n = 29)	(n = 544)
Hypertensive disorders^a^	127	4	91	8	230 (42%)
Postpartum haemorrhage	48	5	13	3	69 (13%)
Other obstetric causes^b^	31	18	3	8	60 (11%)
Placenta complications^c^	46	5	2	1	54 (9.9%)
Ruptured uterus	26	0	7	2	35 (6.4%)
Extrauterine pregnancy	14	0	14	1	29 (5.3%)
Obstetric infections^d^	12	6	2	2	22 (4.0%)
Abortions < 28 weeks^e^	11	3	6	1	21 (3.9%)
Indirect causes^f^	8	7	2	3	20 (3.7%)
Other causes < 28 weeks^g^	3	0	1	0	4 (0.74%)

^a^Severe pre-eclampsia and eclampsia (212 MNM, 11 deaths), HELLP syndrom (Hemolysis Elevated Liver Enzymes Low Platelet count) (6 MNM, 1 death).

^b^Iatrogenic complications (9 MNM, 5 deaths), anaemia (12 MNM, 4 deaths), peripartum cardiomyopathy (9 MNM, 9 deaths), intra-abdominal haemorrhage after CS (2 MNM, 2 deaths), thrombo-embolic events (3 deaths), intoxication by herbs taken by woman to augment labour (2 deaths), coagulopathy due to intra-uterine fetal death (1 MNM, 1 death), renal failure (1 MNM).

^c^Ablatio (34 MNM, 6 deaths), previa (5 MNM), accreta (6 MNM), coriocarcinoma (3 MNM).

^d^Gestational age ≥ 28 weeks.

^e^Spontaneous (11 MNM, 2 deaths) and unsafe abortions (6 MNM, 2 deaths).

^f^HIV (3 MNM, 7 deaths), non-obstetric infections (2 MNM, 1 death), malaria (2 MNM), cholecystitis (1 MNM), asthma (1 death), sickle cell anaemia (1 MNM), epilepsy (1 MNM), intracranial lesion (1 death).

^g^Molar pregnancy (3 MNM), hyperemesis (1 MNM).

The total CS rate was 53% at the university hospital and 6.5% at the regional hospital. Among women who experienced MNM events or died and had a gestational age of ≥ 28 weeks, the CS rate was 56% at the university hospital and 20% at the regional hospital. Indications of CSs are presented in Table A3, Additional file 1.

**Figure 1:**
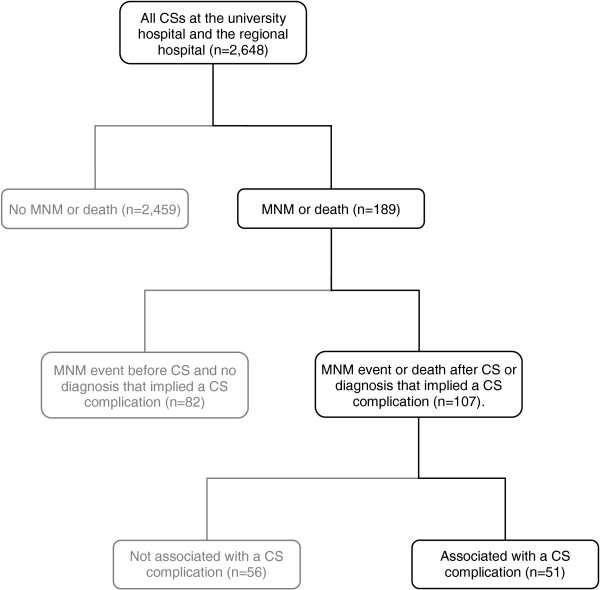
**Flow diagram on inclusion of maternal near-miss (MNM) events and maternal deaths associated with caesarean section (CS) between February and June 2012.** One hundred and eighty-nine women who experienced MNM events or died had undergone CS. Of these, 107 experienced MNM events or died after CS or had a diagnosis that implied a CS complication. After the assessment, we found that 51 cases were associated with a CS complication and 56 cases were caused by other disorders (uterine rupture (n = 22), eclampsia (n = 13), ablation placenta (n = 8), peripartum cardiomyopathy (n = 6), placenta previa or accreta (n = 4), shock due to postpartum haemorrhage with fulfilment of criterion more than seven days after the operation (n = 2), and anaemia (n = 1)).

After assessing all cases that fulfilled their first MNM criterion or died after CS or had a diagnosis that implied a CS complication (n = 107), we found that 49 MNM events and 10 deaths were associated with CS (Figure 1). Of these, 20 had a strong, 27 had a moderate, and 4 had a weak association with the operation. Among the strong associations, 10 were severe infections in the scar, 6 were high spinal anaesthesia causing cardiac arrest, 2 were intra-abdominal bleeding from the CS scar, and 2 were ureter injuries. Cases of moderate association included postpartum haemorrhage leading to shock (n = 12), hysterectomy (n = 7), death (n = 3), or transfusion of ≥ 5 units of blood (n = 2). Among those cases having a moderate association were cases of cardiac arrest due to high spinal anaesthesia (n = 2) and intra-abdominal haemorrhage from the uterus scar (n = 1), but those patients also had eclampsia. Weak associations included hysterectomy or blood transfusion due to postpartum haemorrhage with pre-existing conditions.

Table 4 presents the MNM events and deaths associated with CS complications. CS complications accounted for 7.9% (95% CI 5.6–11) of MNM and 13% (95% CI 6.4–23) of maternal deaths. The risk of experiencing a life-threatening CS complication was three times higher at the regional hospital (22 per 1,000 operations) than the university hospital (7.0 per 1,000 operations) (risk ratio 3.2, 95% CI 1.5–6.6).

**Table 4:** **Estimated risk of a life-threatening CS complication per 1,000 operations at a university hospital and a regional hospital in Tanzania between February and June 2012, including 95% confidence intervals**

	Delivered at the university hospital	Delivered at the regional hospital	Delivered at other institutions ^a^	Total
MNM^b^				
Number associated with CS	12	11	14	37
Risk/1,000 operations^c^	6.0 (3.1–10)	17 (8.7–31)	N/D^d^	N/D^d^
Maternal deaths				
Number associated with CS	2	3	5	10
Risk/1,000 operations^c^	1.0 (0.1–3.6)	4.7 (1.0–14)	N/D^d^	N/D^d^
MNM^b^ and deaths				
Number associated with CS	14	14	19	47
Risk/1,000 operations^c^	7.0 (3.8–12)	22 (12–37)	N/D^d^	N/D^d^

^a^Delivered at other institutions and referred to the university hospital or the regional hospital.

^b^Maternal near-miss.

^c^Number of cases with strong or moderate association with CS divided by the total number of CS at the university hospital (n = 2,014) and the regional hospital (n = 634).

^d^No denominator available for total number of CSs at other institutions.

## Discussion

As one of the first studies applying the WHO criteria in a low-resource setting [9–11], we have identified a high occurrence of MNM and death at a university hospital and a regional hospital in Tanzania. Major causes were eclampsia (42%) and postpartum haemorrhage (13%), but there were also nine MNM events and five deaths due to iatrogenic complications, including high spinal anaesthesia, uterine perforations, ureter injuries, magnesium intoxication, and blood transfusion reaction. CS complications accounted for a large proportion of MNM and death, and the risk per 1,000 operations was high.

### Strengths and limitations

Since the patient uptake and resources at the two hospitals we investigated are comparable to other hospitals in the region, our results may be transferrable to similar settings. The WHO criteria allowed us to identify women with direct signs and symptoms of organ dysfunction, which more reliably reflects a life-threatening condition than using a diagnosis-based inclusion [4]. The data collection was performed in a structured and consistent manner. Having the same doctor review all medical records prevented different interpretations of the criteria and causes. When assessing cases potentially associated with CS, we considered each one individually, taking into account other factors that might have contributed to the MNM event or death, such as pre-existing conditions and the indication of the operation. By doing so, we hoped to identify adverse outcomes attributed to the CS per se. The manuscript follows the Strengthening the Reporting of Observational Studies (STROBE) recommendations (Table A4, Additional file 2).

Our study has limitation that needs further discussion. The applicability of the WHO criteria in low-resource settings has recently been questioned in two studies from Malawi [9] and Tanzania [26]. As described above, we could not apply all WHO criteria due to limited resources at the facilities. Since some women had severe complications, yet did not fulfill any clinical criteria, we might have underestimated the MNMR, especially at the regional hospital. At the time we started the study, there were no studies published that applied the new criteria. When we compare our results with recently published work, we can see that other researchers have made a different interpretation of the criterion “uncontrollable fits”, only including women with continuous seizures [11, 26]. This has to be considered when comparing our estimated MNMR with other studies. As we did not screen wards outside the obstetric and gynaecological departments, some pregnant women admitted to other wards potentially could have been missed. We believe, however, that this had little impact on the results, since most pregnant women in these settings were admitted to the obstetric and gynaecological wards, even if they experienced other medical problems such as malaria, HIV, cholecystitis, and postpartum psychosis. Due to practical reasons, we could not follow women after their discharge from hospital. Since some women may have died at home or at another institution within 42 days, we might have underestimated the number of maternal deaths. As autopsies were not performed, the underlying cause of death was based solely on information in the medical record, and must therefore be interpreted with caution.

### Occurrence of MNM and death

The occurrence of MNM in our study was considerably higher than reports from middle-income countries [5–7], but agreed with other research from Africa: a study in a rural referral hospital in northern Tanzania found an MNMR of 23.6 per 1,000 live births [11], and one from a tertiary facility in urban Ghana estimated the MNMR to be 28.6 per 1,000 live births [10]. The high MNMR of 92 per 1,000 live births at the university hospital in our study is probably due to more referred patients (87% compared to between 20.9% to 64.4% in the other studies), but may also be explained by different interpretations of the criteria, as described above. The higher morality index of 12.9%–18% in our and similar studies from Africa [10, 11], compared to 10.4%–11.1% in studies from middle-income countries [6, 7], demonstrates that a larger proportion of critically ill women in low-resource settings die from their complications.

### Panorama of MNM and death

The panorama of MNM and death highlights several areas in need of attention. Although eclampsia and postpartum haemorrhage are known as major causes of MNM and death [1], and efforts are continuously made to reduce their incidence, they accounted for the largest proportion of severe illness in our study. In spite of a 100% antenatal care coverage in Dar es Salaam [22], hypertension or pre-eclampsia were detected at the antenatal clinic in only 16% of the women who experienced MNM events or died, indicating that the antenatal controls have been insufficient to detect these risk pregnancies. Many women suffered from severe complications such as hysterectomy due to postpartum haemorrhage, even though active management of third stage of labour is promoted at most facilities. The finding that 71% of women with uterine ruptures met their first MNM criterion after arrival undoubtedly raises questions about surveillance during labour. Also, iatrogenic complications involving intravenous infusions and injuries during surgery indicate that there is a need to promote patient safety at these settings.

### Risks with CS

Although the risks with CS in middle- and low-resource settings have been described before [16, 17], there is to our knowledge no studies from low-income countries estimating the proportion of MNM and death directly attributed to CS complications or risk per 1,000 operations. Our finding that CS complications accounted for 13% of maternal deaths is coherent with our previous result of an increase in the CS rate accompanied by an increase in the MMR at the university hospital during the last decade [15]. MNM and deaths attributed to CS complications are especially worrying in the light of rapidly rising CS rates in many low-income countries [12, 15, 17, 27]. Reports that CSs are performed on non-medical indications [27, 28] and among low-risk groups [15] raise concerns about unnecessary morbidity and mortality after CS. Our estimate of the risk of death due to CS complications (between 1.0 and 4.7 per 1,000 operations) compared to a study from the US (0.0087 per 1,000 operations) [20] illustrates the danger CS might constitute in low-resource settings [13].

### Distribution of resources

The uneven distribution of human and material resources between different health care facilities in the Dar es Salaam region most likely contributed to the high burden of MNM and death found in our study. Although peripheral hospitals receive the majority of patients, their ability to provide adequate maternal care is restricted due to the extensive lack of drugs, sterile packs, postoperative beds, blood for transfusion, electricity, and trained staff. In our study, 87% of MNM events and deaths at university hospital were referred. The referral system in Dar es Salaam is, however, ineffective, and delays might aggravate an already severe medical condition. With a higher standard of care at the peripheral hospitals, many MNM events and deaths might have been prevented.

The resource shortage at peripheral hospitals might also explain the elevated risk of CS complications at the regional hospital compared to the university hospital. As described before, a large proportion of surgery and anaesthesiology at the peripheral hospitals is provided by assistant medical officers instead of medical doctors. Although previous studies have not detected an increased risk of maternal death or other complications if an assistant medical officer, rather than a medical doctor, performs the surgery [13, 23, 29], untrained anaesthesiology staff has been associated with an increased risk of maternal death during or after CS [13]. Insufficient training of anaesthesiology staff might explain some of the MNM events and deaths in our study, such as the cases of high spinal anaesthesia. The occurrence of postpartum haemorrhage and infections after CS, raises concerns about postoperative surveillance and use of prophylactic antibiotics at the peripheral hospitals.

### Clinical implications

Based on our findings, we suggest measures that might reduce the incidence of MNM and death. As most women with eclampsia fulfilled MNM criteria on arrival, antenatal services need to more effectively detect women at risk for hypertensive disorders and urge them to seek health care early when signs and symptoms arise. Magnesium sulphate should be readily available at health centres and smaller hospitals, since a majority of eclampsia cases were referrals. Measures to decrease the number of severe complications related to postpartum haemorrhage, including emergency hysterectomy, should be undertaken, such as closer monitoring, better availability of utero-tonic drugs, and enforcement of the use of uterine artery ligation and B-lynch suture. Also, surveillance and active management during labour needs to be improved, especially at peripheral hospitals, in order to detect and prevent uterine ruptures. Auditing cases with iatrogenic complications might help to identify risk situations and strengthen patient safety.

We identified an urgent need to decrease the maternal risks associated with CS. As this is most effectively done by avoiding unnecessary CSs, auditing CS indications, introducing a mandatory second opinion for CS decisions, and promoting active management of labour can be appropriate measures [30]. In many hospitals, high CS rates are accompanied by an underuse of instrumental deliveries [15, 28]. Therefore, increasing the use of instrumental deliveries might be another way to avoid unnecessary CSs. Also, safety during and after the procedure needs to be improved, especially at peripheral hospitals. This includes more training of staff in order to avoid anaesthetic and surgical complications, strict use of prophylactic antibiotics to decrease the number of postoperative infection, as well as earlier detection and more effective treatment of postpartum haemorrhage after CS.

## Conclusion

We have identified a high occurrence of MNM and death in the Dar es Salaam area. Major causes were hypertensive disorders and postpartum haemorrhage, but we also detected several cases of iatrogenic complications. CS complications accounted for a high proportion of MNM and death. The maternal risks of CS in low-resource settings must not be overlooked, and measures should be taken to avoid unnecessary CSs. More comprehensive training of staff, improved postoperative surveillance, and a more equal distribution of resources within the health care system might reduce the risks of CS complications, especially at peripheral hospitals. Our definition of MNM and death associated with CS complications enabled us to estimate the risks of CS in this setting and might be useful in future studies.

## Electronic supplementary material

Additional file 1: Table A1: Criteria used for inclusion of maternal near-miss events, including definitions, interpretations, and applicability at the two settings. **Table A2.** Criteria met among women with maternal near-miss morbidity at a university hospital and a regional hospital in Tanzania between February and June 2012. **Table A3.** Indications of caesarean section (CS) among women with maternal near-miss and death at a university hospital and regional hospital in Tanzania between February and June 2012. (DOC 115 KB)

Additional file 2: Table A4: STROBE checklist of items that should be included in reports of cross-sectional studies. (DOC 94 KB)

## Abbreviations

MNM: Maternal near-miss
WHO: World Health Organization
CS: Caesarean section
MMR: Maternal mortality ratio
CI: Confidence interval
MNMR: Maternal near-miss ratio.
