# Supplementary material for: Maternal near-miss and death and their association with caesarean section complications: a cross-sectional study at a university hospital and a regional hospital in Tanzania
Source: BMC Pregnancy Childbirth. 2014 Jul 23;14:244. doi: 10.1186/1471-2393-14-244 (PMC4133054; doi:10.1186/1471-2393-14-244)
Supplement: Supplementary file 1 — Additional file 1: Table A1: Criteria used for inclusion of maternal near-miss events, including definitions, interpretations, and applicability at the two settings. Table A2. Criteria met among women with maternal near-miss morbidity at a university hospital and a regional hospital in Tanzania between February and June 2012. Table A3. Indications of caesarean section (CS) among women with maternal near-miss and death at a university hospital and regional hospital in Tanzania between February and June 2012. (DOC 115 KB) [file 12884_2013_1132_MOESM1_ESM.doc]

Table A1. Criteria used for inclusion of maternal near-miss events, including definitions, interpretations, and applicability at the two settings.

_________________________________________________________________________________

|  |  |  |  |
| --- | --- | --- | --- |
| Criteria | Definition according to WHO [2,25] | Applicable at the University Hospital | Applicable at the Regional Hospital |
|  |  |  |  |
| **Clinical** |  |  |  |
| Acute cyanosis | Blue or purple colouration of the [skin](http://en.wikipedia.org/wiki/Skin) or [mucous membranes](http://en.wikipedia.org/wiki/Mucous_membrane) due to low oxygen saturation^a^ | Yes | Yes |
|  |  |  |  |
| Gasping | Terminal respiratory pattern, the breath is convulsively and audibly caught. | Yes | Yes |
|  |  |  |  |
| Severe bradypnea | Respiratory rate < 6 | Yes | Yes |
|  |  |  |  |
| Severe tachypnea | Respiratory rate > 40 | Yes | Yes |
|  |  |  |  |
| Shock | Persistent systolic blood pressure ≤ 80 mmHg or a persistent systolic blood pressure ≤ 90 mmHg with a pulse rate ≥ 120. | Yes | Yes |
|  |  |  |  |
| Oliguria | Urinary output < 30 ml/hour for 4 hours or < 400 ml/24 hours non-responsive to fluids or diuretics. | Yes | Yes |
|  |  |  |  |
| Failure to form clots | Bedside clotting test^b^ or absence of clotting from the IV site after 7 minutes | Yes | Yes^c^ |
|  |  |  |  |
| Prolonged unconsciousness | Complete or near-complete lack of responsiveness  to external stimuli. | Yes | Yes |
|  |  |  |  |
| Cardiac arrest | Sudden absence of pulse and loss of consciousness. | Yes | Yes |
|  |  |  |  |
| Stroke | Neurological deficit of cerebrovascular cause persisting ≥ 24 hours. | Yes | Yes |
|  |  |  |  |
| Uncontrollable fits | Refractory, persistent convulsions^d^ or status epilepticus. | Yes | Yes |
|  |  |  |  |
| Total paralysis | Complete or partial paralysis of both sides of the body. | Yes | Yes |
|  |  |  |  |
| Jaundice in the presence of pre-eclampsia | Presence of hypertension (blood pressure > 140/90) associated with proteinuria (> 1 + dipstick in ≥ 2 samples) and jaundice. | Yes | Yes |
|  |  |  |  |
| Criteria | Definition according to WHO[1, 2] | Applicable at the University Hospital | Applicable at the Regional Hospital |
| **Laboratory-based** |  |  |  |
| Severe hypoxemia | Oxygen saturation < 90% for ≥ 60 minutes | Yes | No |
|  |  |  |  |
| Severe hypoxemia | PaO2/FiO2 < 200 mmHg | No | No |
|  |  |  |  |
|  |  |  |  |
| Acute severe azotemia | Creatinine > 300 mmol/l or > 3,5 mg/dl | Yes | No |
|  |  |  |  |
| Severe acute  hyperbilirubinemia | Bilirubin > 100 μmol/l or > 6.0 mg/dl. | Yes | No |
|  |  |  |  |
| Severe acidosis | pH < 7.1 | No | No |
|  |  |  |  |
| Severe hypoperfusion | Lactate > 5 mmol/l or > 45 mg/dl | No | No |
|  |  |  |  |
| Severe acute thrombocytopenia | <50 000 platelets/ml | Yes | No |
|  |  |  |  |
| Metabolic coma | Loss of consciousness and glucose + ketoacids in urine. | Yes | No |
|  |  |  |  |
| **Management-based** |  |  |  |
| Use of continuous vasoactive drugs | Uninterrupted infusion of dopamine, epinephrine, or norepinephrine. | Yes | No |
|  |  |  |  |
| Hysterectomy | Surgical removal of the uterus following infection or haemorrhage. | Yes | Yes |
|  |  |  |  |
| Massive transfusion | Transfusion  of ≥ 5 units of blood^e^ | Yes | Yes^f^ |
| Intubation and ventilation not related to anaesthesia |  | Yes | Yes |
|  |  |  |  |
| Dialysis for acute renal failure |  | No^g^ | No^g^ |
|  |  |  |  |
| Cardiopulmonary  resuscitation | Emergency procedures including chest compressions and lung ventilation. | Yes | Yes |

_________________________________________________________________________________

^a^As defined by the authors.

^b^The bedside clotting test: (1) Take 2 m l of venous blood into a small, dry clean glass-tube; (2) Hold the tube in your closed fist to keep it warm; (3) After 4 minutes, tip the tube slowly to see if a clot is forming. Then tip it again until the blood clots and tube can be turned upside down; (4) Failure for a clot o form after 7 minutes or a soft clot that breaks down suggests coagulopathy.

^C^Could have been applicable given the resources, but was not regularly practiced.

^d^No further explanation provided in the guidelines. Was interpreted as loss of consciousness and repeated fits.

^e^Including all types of blood products.

^f^Blood for transfusion was available but seldom in these quantities.

^g^Not available at the two hospitals at the time of the study, but was available at private hospitals in Dar es Salaam.

Table A2. Criteria met among women with maternal near-miss morbidity at a university hospital and a regional hospital in Tanzania between February and June 2012

___________________________________________________________________________

|  |  |  |  |
| --- | --- | --- | --- |
| Criteria | University hospital | Regional  hospital | Total |
|  |  |  |  |
| **Clinical** |  |  |  |
| Gasping | 2 (0.43%) | 0 | 2 (0.33%) |
| Severe bradypnea or tachypnea | 18 (3.8%) | 2 (1.4%) | 20 (3.3%) |
| Shock | 108 (23%) | 41 (28%) | 149 (24%) |
| Oliguria | 8 (1.7%) | 0 | 8 (1.3%) |
| Failure to form clots | 18 (3.8%) | 0 | 18 (2.9%) |
| Prolonged unconsciousness | 10 (2.1%) | 3 (2.1%) | 13 (2.1%) |
| Cardiac arrest | 5 (1.1%) | 0 | 5 (0.81%) |
| Stroke | 9 (1.9%) | 1 (0.68%) | 10 (1.6%) |
| Uncontrollable fits | 124 (26%) | 89 (61%) | 213 (35%) |
| Jaundice in the presence of pre-eclampsia | 5 (1.1%) | 1 (0.68%) | 6 (0.98%) |
|  |  |  |  |
| **Laboratory-based** |  |  |  |
| Severe hypoxemia | 3 (0.64%) | N/A^a^ | 3 (0.49%) |
| Acute severe azotemia | 19 (4.1%) | N/A^a^ | 19 (3.1%) |
| Severe acute hyperbilirubinemia | 4 (0.85%) | N/A^a^ | 4 (0.65%) |
| Severe acute thrombocytopenia | 26 (5.5%) | N/A^a^ | 26 (4.2%) |
|  |  |  |  |
|  |  |  |  |
| **Management-based** |  |  |  |
| Hysterectomy | 54 (12%) | 9 | 63 (10%) |
| Transfusion for ≥ 5 U of blood | 47 (10%) | 0 | 47 (6.7%) |
| Ventilation > 60 minutes | 2 (0.43%) | 0 | 2 (0.33%) |
| Dialysis for acute renal failure | 2 (0.43%) | N/A^a^ | 2 (0.33%) |
| Cardio-pulmonary resuscitation | 5 (1.1%) | 0 | 5 (0.81%) |
|  |  |  |  |
| Total | 467 (100%) | 146 (100%) | 615^b^ (100%) |

___________________________________________________________________________

^a^Not Applicable due to shortage of resources

^b^377 women fulfilled one criterion, 90 women fulfilled two or more criteria. MNM criteria “cyanosis” and “continuous use of vaso-active drugs” were fulfilled by some cases, but these women passed away during the observation period.

Table A3. Indications of caesarean section (CS) among women with maternal near-miss and death at a university hospital and regional hospital in Tanzania between February and June 2012

___________________________________________________________________________

| CS indication | Cases not associated with CS | Cases associated with CS | Total |
| --- | --- | --- | --- |
|  |  |  |  |
| Obstructed labor | 25 | 22 | 47 (25%) |
| Previous scar | 13 | 11 | 24 (13%) |
| Eclampsia | 18 | 2 | 20 (11%) |
| Failed induction | 17 | 0 | 17 (9.0%) |
| Ablatio placenta | 13 | 0 | 13 (6.9%) |
| Multiple pregnancy | 10 | 2 | 12 (6.3%) |
| No indication stated | 5 | 5 | 10 (5.3%) |
| Fetal distress | 6 | 2 | 8 (4.2%) |
| Placenta previa | 4 | 1 | 5 (2.6%) |
| Breech | 2 | 1 | 3 (1.6%) |
| Cord prolapse | 1 | 1 | 2 (1.1%) |
| Other | 0 | 1 | 1 (0.5%) |
|  |  |  |  |
| Total | 138 | 51 | 189^a^ (100%) |

___________________________________________________________________________

^a^ Three listed as elective CSs and 186 listed as emergency CSs.
