# Supplementary material for: Maternal near-miss and death and their association with caesarean section complications: a cross-sectional study at a university hospital and a regional hospital in Tanzania
Source: BMC Pregnancy Childbirth. 2014 Jul 23;14:244. doi: 10.1186/1471-2393-14-244 (PMC4133054; doi:10.1186/1471-2393-14-244)
Supplement: Supplementary file 2 — Additional file 2: Table A4: STROBE checklist of items that should be included in reports of cross-sectional studies. (DOC 94 KB) [file 12884_2013_1132_MOESM2_ESM.doc]

Table A4. STROBE checklist of items that should be included in reports of cross-sectional studies.

|  | Item No | Recommendation | Authors’ comments |
| --- | --- | --- | --- |
| **Title and abstract** | 1 | (*a*) Indicate the study’s design with a commonly used term in the title or the abstract | The study design (cross-sectional) is indicated in the title. |
|  |  | (*b*) Provide in the abstract an informative and balanced summary of what was done and what was found | The abstract is structured to fit the requirements of the journal and has been prepared to provide an informative summary of the study. |
| Introduction | | |  |
| Background/rationale | 2 | Explain the scientific background and rationale for the investigation being reported | In the “Background”, we explain the maternal near-miss (MNM) concept, that few studies have been conducted in low-income countries, the global increase in caesarean section (CS) rates, the maternal risks with CS, and the problems that exist with assessing maternal risks with CS. |
| Objectives | 3 | State specific objectives, including any pre-specified hypotheses | The aim of the study and our specific objectives are presented in the last paragraph of the “Background”. |
| Methods | | |  |
| Study design | 4 | Present key elements of study design early in the paper | “Cross-sectional study” is mentioned in the first sentence of the “Method”. |
| Setting | 5 | Describe the setting, locations, and relevant dates, including periods of recruitment, exposure, follow-up, and data collection | This information is presented in the “Method”. |
| Participants | 6 | (*a*) Give the eligibility criteria, and the sources and methods of selection of participants | The criteria used for inclusion and methods of selection of participants are presented under “Participants” in the “Method” and in Table A1 in Additional file 1. |
| Variables | 7 | Clearly define all outcomes, exposures, predictors, potential confounders, and effect modifiers. Give diagnostic criteria, if applicable | The outcomes (MNM and maternal death) are explained under “Participants” in the “Method”, where we also present references to more information on the MNM concept and definitions of maternal death. Criteria used are presented under “Participants” in the “Method” and in Table A1, Additional file 1. Definitions of MNM or death associated with CS are also presented under “Participants” in the “Method”. |
| Data sources/ measurement | 8* | For each variable of interest, give sources of data and details of methods of assessment (measurement). Describe comparability of assessment methods if there is more than one group | Medical records, antenatal cards, and report books were used as sources of data. Details of assessment and sources of data are presented under “Data collection” and “Participants” in the “Method”. |
| Bias | 9 | Describe any efforts to address potential sources of bias | Cases were followed during their whole hospitalization until discharge in order to record any additional diagnoses and criteria fulfilled (“Participants” in the “Method”). The same researcher included all cases, which prevented different interpretations of the criteria (“Data collection” in the “Method”). Causes of MNM or death were discusses between three of the authors (“Data collection” in the “Method”). When deciding if cases were associated with a CS complication or not, each case was reviewed by the research group in order to reach consensus, and the indication of CS was taken into consideration in order to avoid indication bias (“Participants” in the “Method”). |
| Study size | 10 | Explain how the study size was arrived at | We included all women with MNNM or death between February and June 2012 (“Data collection” in the “Method”). This time period was chosen as we, based on previous experiences of MNM-studies, found it likely to include an adequate number of cases to make our analysis. |
| Quantitative variables | 11 | Explain how quantitative variables were handled in the analyses. If applicable, describe which groupings were chosen and why | The analyses are explained under “Analyses” in the “Method”. |
| Statistical methods | 12 | (*a*) Describe all statistical methods, including those used to control for confounding | The analyses are explained under “Analyses” in the “Method”. |
|  |  | (*b*) Describe any methods used to examine subgroups and interactions | How we examined the subgroup of women that had experienced MNM or death associated with CS is explained under “Participants” in the “Method” and in the second paragraph under “Analyses in the “Method”. |
|  |  | (*c*) Explain how missing data were addressed | Women with missing information on time of discharge were included as MNM cases (First paragraph of “Results”). |
|  |  | (*d*) If applicable, describe analytical methods taking account of sampling strategy | x |
|  |  | (*e*) Describe any sensitivity analyses | x |
| Results | | |  |
| Participants | 13* | (a) Report numbers of individuals at each stage of study—eg numbers potentially eligible, examined for eligibility, confirmed eligible, included in the study, completing follow-up, and analysed | Total number of deliveries and live births (potentially eligible and examined for eligibility) are presented in the first paragraph of the “Results” and in Table 2. The number confirmed eligible and included are presented in the first paragraph of the “Results” and Table 2. In the same paragraph, we also present the number lost to follow-up and that some MNM events were analysed as two separate events.  Number of total CSs, how many were examined for eligibility (MNM or death associated with CS), how many were confirmed eligible (included), and analysed is presented in the seventh paragraph f the “Results” and in Figure 1. |
|  |  | (b) Give reasons for non-participation at each stage | Reasons for why cases were assessed as not being associated with CS are presented in the legend of Figure 1. |
|  |  | (c) Consider use of a flow diagram | Flow diagram of inclusion of cases associated with CS complications is presented in Figure 1. |
| Descriptive data | 14* | (a) Give characteristics of study participants (eg demographic, clinical, social) and information on exposures and potential confounders | This data is presented in Table 1. |
|  |  | (b) Indicate number of participants with missing data for each variable of interest | This data is presented in Table 1. |
| Outcome data | 15* | Report numbers of outcome events or summary measures | This data is presented in Table 2 and Table 4. |
| Main results | 16 | (*a*) Give unadjusted estimates and, if applicable, confounder-adjusted estimates and their precision (eg, 95% confidence interval). Make clear which confounders were adjusted for and why they were included | This data is presented in Table 2 and Table 4. |
|  |  | (*b*) Report category boundaries when continuous variables were categorized | This data is presented in Table 1. |
|  |  | (*c*) If relevant, consider translating estimates of relative risk into absolute risk for a meaningful time period | Risk per 1,000 operations of experiencing a MNM event or death associated with CS is presented in Table 4. |
| Other analyses | 17 | Report other analyses done—eg analyses of subgroups and interactions, and sensitivity analyses | Analyses of cases associated with CS are presented on the seventh and eight paragraph of the “Results”. |
| Discussion | | |  |
| Key results | 18 | Summarise key results with reference to study objectives | Summary is presented in the first paragraph of the “Discussion”. |
| Limitations | 19 | Discuss limitations of the study, taking into account sources of potential bias or imprecision. Discuss both direction and magnitude of any potential bias | Limitations and direction of potential bias are presented in the third paragraph of the “Discussion”. |
| Interpretation | 20 | Give a cautious overall interpretation of results considering objectives, limitations, multiplicity of analyses, results from similar studies, and other relevant evidence | Interpretations of the results are presented in the fourth to tenth paragraph of the “Discussion”. |
| Generalisability | 21 | Discuss the generalisability (external validity) of the study results | Generalisability is discussed in the second paragraph of the “Discussion” and in the “Conclusion”. |
| Other information | | |  |
| Funding | 22 | Give the source of funding and the role of the funders for the present study and, if applicable, for the original study on which the present article is based | Funding is presented in “Acknowledgements”. |
